# Association of distinct type 1 bone morphogenetic protein receptors with different molecular pathways and survival outcomes in neuroblastoma

**DOI:** 10.1042/NS20200006

**Published:** 2020-04-23

**Authors:** Amnah M. Alshangiti, Sean L. Wyatt, Erin McCarthy, Louise M. Collins, Shane V. Hegarty, Aideen M. Sullivan, Gerard W. O’Keeffe

**Affiliations:** 1 Department of Anatomy and Neuroscience, University College Cork (UCC), Cork, Ireland; 2 Cork Neuroscience Centre, University College Cork, Cork, Ireland; 3 School of Biosciences, Cardiff University, Museum Avenue, Cardiff, U.K.

**Keywords:** BMPR, bone morphogenetic protein, neurite growth, Neuroblastoma, Smad, survival

## Abstract

Neuroblastoma (NB) is a paediatric cancer that arises in the sympathetic nervous system. Patients with stage 4 tumours have poor outcomes and 20% of high-risk cases have *MYCN* amplification. The bone morphogenetic proteins (BMPs) play roles in sympathetic neuritogenesis, by signalling through bone morphogenetic protein receptor (BMPR)2 and either BMPR1A or BMPR1B. Alterations in BMPR2 expression have been reported in NB; it is unknown if the expression of BMPR1A or BMPR1B is altered. We report lower *BMPR2* and *BMPR1B*, and higher *BMPR1A*, expression in stage 4 and in *MYCN*-amplified NB. Kaplan–Meier plots showed that high *BMPR2* or *BMPR1B* expression was linked to better survival, while high *BMPR1A* was linked to worse survival. Gene ontology enrichment and pathway analyses revealed that *BMPR2* and *BMPR1B* co-expressed genes were enriched in those associated with NB differentiation. *BMPR1A* co-expressed genes were enriched in those associated with cell proliferation. Moreover, the correlation between *BMPR2* and *BMPR1A* was strengthened, while the correlation between *BMPR2* and *BMPR1B* was lost, in *MYCN*-amplified NB. This suggested that differentiation should decrease *BMPR1A* and increase *BMPR1B* expression. In agreement, nerve growth factor treatment of cultured sympathetic neurons decreased *Bmpr1a* expression and increased *Bmpr1b* expression. Overexpression of dominant negative BMPR1B, treatment with a BMPR1B inhibitor and treatment with GDF5, which signals via BMPR1B, showed that BMPR1B signalling is required for optimal neuritogenesis in NB cells, suggesting that loss of *BMPR1B* may alter neuritogenesis. The present study shows that expression of distinct BMPRs is associated with different survival outcomes in NB.

## Introduction

Neuroblastoma (NB) is a paediatric cancer that arises in the sympathetic nervous system and is responsible for 15% of all pediatric cancer mortality [1]. The International Neuroblastoma Staging System (INSS) classifies NB into stages 1–4 and stage 4s [2]. Patients with stage 1 or 2 tumours have an excellent prognosis, while those with stage 3 or 4 tumours have a particularly poor outcome. Approximately 20% of the high-risk NB tumours present with amplification of *MYCN* [3]. While the molecular drivers of NB progression are multi-factorial, a whole-genome analysis of 87 NB samples identified frequent alterations in the expression of genes associated with neuritogenesis, in high-risk tumours [3]. This suggests that alterations in the molecular mechanisms that control normal neuritogenesis may occur in cells of the sympathetic lineage in NB.

One key group of signals that control sympathetic development is the bone morphogenetic proteins (BMPs), the largest subgroup of the transforming growth factor (TGF)-β superfamily of ligands [4]. BMP ligands signal by binding a heteromeric complex of bone morphogenetic protein receptor (BMPR)2 and one of two type 1 BMPRs, BMPR1A and BMPR1B, which are transmembrane serine/threonine kinase receptors [5]. Individual BMP ligands have different affinities for BMPR complexes. For example, BMP2, BMP7 and growth/differentiation factor (GDF)5 (also known as BMP14) all bind to BMPR2 [6,7]. However, BMP2 and BMP7 bind to BMPR1A or BMPR1B as the second receptor in the complex [8], while GDF5 binds with high affinity to BMPR1B, but not to BMPR1A [9,10]. In the canonical BMP pathway BMP receptors activate transcription factors known as the R-Smads (Smad1/5/8), which form a complex with Smad4, which translocates to the nucleus to regulate gene expression [4]. BMPRs can also activate a number of non-canonical pathways, including MAPK and PI3K pathways, depending on the cellular context [11–13].

It is well established that BMPs and BMPRs play key roles in the development of sympathetic neurons [14,15]. For example, BMPs acting through BMPRs have been shown to promote the specification and dendritic growth of sympathetic neurons [16–19], while GDF5 controls sympathetic axon growth and target innervation [20]. Given that BMP signalling controls neuritogenesis in the developing sympathetic nervous system, alterations in expression of the BMPRs may contribute to the defects in neuritogenesis seen in NB [3]. In support of this, a previous study has shown that there is a significant down-regulation of BMPR2 expression in NB, that BMPR2 knockdown increased cell growth and tumourigenicity in mice, and that patients with high BMPR2 expression have better overall survival [21]. It has also been shown that Smad4 is required for growth inhibition, invasion and metastasis of NB cells, and that patients with high Smad4 expression have greater survival probability [22]. Moreover, several BMPs have been shown to promote the neurite growth and differentiation of human NB cells; these include BMP4 [23], BMP9 [24], BMP2 [25], BMP6 [26] and GDF5 [27]. There is little knowledge on any alterations of BMPR1A and BMPR1B expression that may occur in NB, nor is it known whether the expression of these receptors is associated with NB patient outcomes and/or neuritogenesis.

## Methods

### Gene expression analysis of human NB samples

Gene expression data of human NB samples from GSE: 62564 [28–30], GSE: 45547 [31] and GSE: 120572 [32] data sets were analysed. Expression data and Kaplan–Meier plots were generated using the R2: Genomics Analysis and Visualisation Platform (http://r2.amc.nl). For gene co-expression analysis, all genes that displayed a significant correlation with *BMPR2, BMPR1A* or *BMPR2* expression after a Bonferroni multiple testing correction were analysed. Gene ontology (GO) and Panther pathway enrichment analysis was carried out using tools available at www.geneontology.org. All gene expression data are presented as mean rank expression values. Differences in expression were analysed using a Kruskal–Wallis test with a Dunn’s post-hoc test, or Mann–Whitney test, as appropriate.

### Quantitative real-time PCR (RT-qPCR)

The levels of *Bmpr1a, Bmpr1b* and *Bmpr2* mRNAs in postnatal day (P)0 mouse sympathetic neurons were quantified by RT-qPCR relative to a geometric mean of reference mRNAs for the three housekeeping enzymes, glyceraldehyde phosphate dehydrogenase (*Gapdh*), succinate dehydrogenase (*Sdha*) and hypoxanthine phosphoribosyltransferase-1 (*Hprt1*). Briefly, 5 μl of total RNA from cultured sympathetic neurons was reverse transcribed for 1 h at 45°C using the AffinityScript kit (Agilent Technologies, Berkshire, United Kingdom) in a 25 μl reaction volume, according to the manufacturer’s instructions. About 2 μl of cDNA was amplified in a 20 μl reaction volume using Brilliant III ultrafast qPCR master mix reagents (Agilent Technologies) with 150 nM of primers and 300 nM of dual-labeled (FAM/BHQ1) hybridization probes specific to each of the cDNAs (MWG/Eurofins, Ebersberg, Germany) using the Mx3000P platform (Agilent Technologies). The PCR primers were: *Bmpr1b* forward: 5′-AGT GTA ATA AAG ACC TCC A-3′ and reverse: 5′-AAC TAC AGA CAG TCA CAG-3′; *Bmpr1a* forward: 5′-TAC GCA GGA CAA TAG AAT-3′ and reverse: 5′-AAC TAT ACA GAC AGC CAT-3′; *Gapdh* forward: 5′-GAG AAA CCT GCC AAG TAT G-3′ and reverse: 5′-GGA GTT GCT GTT GAA GTC-3′; *Bmpr2* forward: 5′-ACT AGA GGA CTG GCT TAT-3′ and reverse: 5′-CCA AAG TCA CTG ATA ACA C-3′; *Sdha* forward: 5′-GGA ACA CTC CAA AAA CAG-3′ and reverse: 5′-CCA CAG CAT CAA ATT CAT-3′; *Hprt1* forward: 5′-TTA AGC AGT ACA GCC CCA AAA TG-3′ and reverse: 5′-AAG TCT GGC CTG TAT CCA ACA C-3′. Dual labelled probes were: *Bmpr1b* 5′-FAM-ACC TAC ACC CTA CAC TGC CTC-BHQ1-3′; *Bmpr1a*: 5′-FAM-TGA GCA CAA CCA GCC ATC G-BHQ1-3′; *Bmpr2*: 5′-FAM-CAC AGA ATT ACC ACG AGG AGA-BHQ1-3′; *Gapdh*: 5′-FAM-AGA CAA CCT GGT CCT CAG TGT-BHQ1-3; *Sdha*: 5′-FAM-CCT GCG GCT TTC ACT TCT CT-BHQ1–3, *Hrpt1*: 5′-FAM-TCG AGA GGT CCT TTT CAC CAG CAA G-BHQ1–3′. Levels of *Bmpr1a, Bmpr1b* and reference mRNAs in experimental samples were quantified by reference to a standard curve generated for each of the mRNAs that was constructed from a serial dilution of reverse transcribed adult mouse brain RNA.

### Cell culture and analysis

The present study was carried out in accordance with the principles of the Basel Declaration and within the guidelines of the Home Office Animals (Scientific Procedures) Act, 1986. The protocol was approved by the Cardiff University Ethical Review Board. All patient data that were used were open source data freely available online. NB cells were purchased commercially and did not require ethical approval. Mouse breeding was approved by the Cardiff University Ethical Review Board and was performed within the guidelines of the Home Office Animals (Scientific Procedures) Act, 1986. Sympathetic neurons were dissected from the superior cervical ganglion (SCG) of P0 CD1 mice culled by cervical transection, then trypsinized and plated onto poly-ornithine- and laminin-coated 35 mm tissue culture dishes (Greiner, Gloucestershire, U.K.) in serum-free Ham’s F14 medium supplemented with 0.25% Albumax I (Invitrogen, Paisley, U.K.), as described previously at Cardiff University [33]. Neuronal cultures were grown with 0 to 10 ng/ml NGF and 25 μM of pan-caspase inhibitor Boc-D-FMK (Calbiochem) for 24 h before RNA extraction. SH-SY5Y and SK-N-BE(2) cells (ATTC) were cultured in Minimum Essential Medium supplemented with 100 nM L-glutamine, 1% penicillin–streptomycin, 1% (1:1) non-essential amino acid solution: Ham’s F-12, and 15% foetal bovine serum (FBS). All cells were cultured at 37°C in a humidified atmosphere with 5% CO_2_. For both primary cultures and cell lines, 2 × 10^5^ cells were plated per well of a 24-well plate and treated for 72 h with 0.5 µM K02288, a highly selective 2-aminopyridine-based BMP signalling inhibitor, or with GDF5 (0–50 ng/ml) where indicated. For analysis of neurite length, cultures were labelled with the vital fluorescent dye Calcein-AM (1:500; Invitrogen). Imaging was performed with an Olympus IX71 inverted microscope fitted with an Olympus DP70 camera and images were analysed using ImageJ software. For neurite growth and cluster area measurements, ≥180 cells from three experiments were analysed and data are presented as mean ± SEM.

### Transfection of cell cultures

A total of 2 × 10^5^ of SK-N-BE(2) cells per well were transfected with a pSK^+^ mBMPR-1B dominant negative (mBMPR1B-dn) plasmid (a gift from Lee Niswander & Peter ten Dijke; Addgene plasmid # 49530; http://n2t.net/addgene:49530; RRID:Addgene_49530) and a GFP-expressing plasmid, using TransIT-X2 Dynamic Delivery System (Mirus) according to the manufacturer’s instructions. Images of GFP-positive cells were captured using an Olympus IX71 inverted microscope fitted with an Olympus DP70 camera, and neurite length was analysed using ImageJ. For neurite growth measurements, ≥240 cells from three independent experiments were analysed and data are presented as mean ± S.E.M.

### Statistical analysis of cell culture data

Statistical analysis was performed using GraphPad Prism 6 (©2018 GraphPad Software, CA U.S.A.). Statistical differences were analysed using a Student’s *t*-test or one-way ANOVA as appropriate, with post-hoc tests as indicated in the figure legends.

## Results

### Alterations in expression of specific BMPRs based on stage and *MYCN*-status of NB

**Figure 1:**
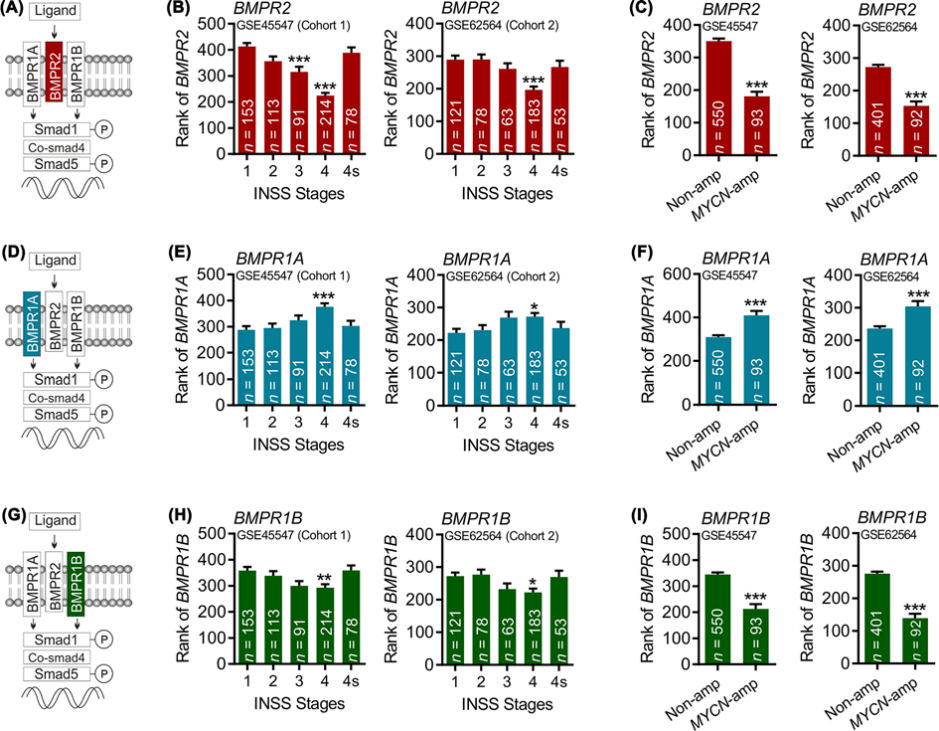
Alterations in expression of specific BMPRs based on stage and *MYCN*-status of NB Schema and graphs showing the mean ranked expression of (**A–C**) *BMPR2*, (**D**–**F**) *BMPR1A* and (**G**–**I**) *BMPR1B*, based on (B,E,H) INNS stage or (C,F,I) *MYCN* status, as indicated, in data set GSE45547 or GSE62564, as indicated (*P<0.05, **P<0.01, ***P<0.001 compared with INNS stage 1 or non-*MYCN*-amplified (‘Non-amp’) NB; Kruskal–Wallis or Mann–Whitney test, as appropriate.

We first sought to examine the expression levels of *BMPR2, BMPR1A* and *BMPR1B* in NB tumour samples. To do this, we probed NB transcriptome data derived from the publicly available data sets, GSE45547 and GSE62564, using the R2 genomics and analysis visualisation platform (https://hgserver1.amc.nl/cgi-bin/r2/main.cgi) with a rank based approach. We first examined the mean ranked expression (hereafter referred to as expression) of the type 2 BMP receptor, BMPR2, and found a significantly lower level of expression of *BMPR2* in stage 4 NB compared with stage 1, in both data sets (Figure 1A,B). We also examined *BMPR2* expression by *MYCN* status, since *MYCN* amplification is the primary and most important prognostic marker of poor survival in NB [34]. We also found that *BMPR2* expression was lower in *MYCN*-amplified NB than in non-*MYCN*-amplified NB in both data sets (Figure 1C). We next examined the expression of the two type 1 BMPRs, BMPR1A and BMPR1B, which are required for signal transduction following ligand binding to BMPR2. In contrast with *BMPR2*, there was a significant increase in the expression of *BMPR1A* in stage 4 NB compared to stage 1 in both data sets (Figure 1D,E). There was also an increase in *BMPR1A* expression in *MYCN*-amplified NB when compared with non-*MYCN*-amplified NB in both data sets (Figure 1F). In contrast with *BMPR1A*, we found that the expression of *BMPR1B* was significantly decreased in stage 4 NB (Figure 1G,H) and in *MYCN*-amplified NB samples (Figure 1I). These data suggest that the expression of distinct BMPRs may be associated with different survival outcomes in NB patients.

### Associations between expression of BMPRs and survival probability in NB patients

**Figure 2:**
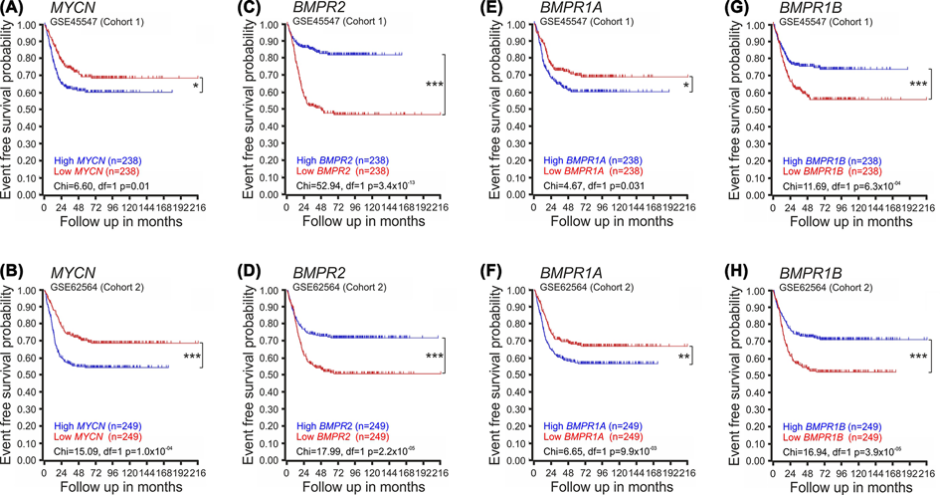
Associations between expression of BMPRs and survival probability in NB patients Kaplan–Meier plots showing the event-free survival probability of patients with ‘high’ or ‘low’ (**A** and **B**) *MYCN*, (**C** and **D**) *BMPR2*, (**E** and **F**) *BMPR1A*, or (**G** and** H**) *BMPR1B* expression divided based on the median expression value of each gene in data set (A,C,E,G) GSE45547 (*n*=476) or (B,D,F,H) GSE62564 (*n*=498). *P<0.05, **P<0.01, *** *P*<0.001; exact *P* values are indicated in black just above the *x*-axis on each graph.

To investigate any associations between BMPR expression and survival probability, we constructed Kaplan–Meier survival plots of the *n*=476 NB cases in the GSE45547 and the *n*=498 NB cases in the GSE62564 data sets. To do this, the samples were divided into two groups that were classified as having ‘high’ or ‘low’ expression *of MYCN* (as a known control) or of *BMPR*, with the cut-off based on the median ranked gene expression value in each data set. As expected, patients with high *MYCN* expression had significantly lower probability of survival (Figure 2A,B). Interestingly, patients with high *BMPR2* expression had a significantly greater survival probability than those with low *BMPR2* expression (Figure 2C,D). In contrast, patients with high *BMPR1A* expression had a significantly lower survival probability than those with low *BMPR1A* expression (Figure 2E,F), while those with high BMPR1B expression had a significantly higher survival probability than those with low BMPR1B expression (Figure 2G,H). This suggests that BMPR1A and BMPR1B are associated with distinct genes in NB cells.

### Specific BMPRs display distinct gene co-expression profiles that are enriched in genes associated with molecular pathways linked to NB proliferation or differentiation

**Figure 3:**
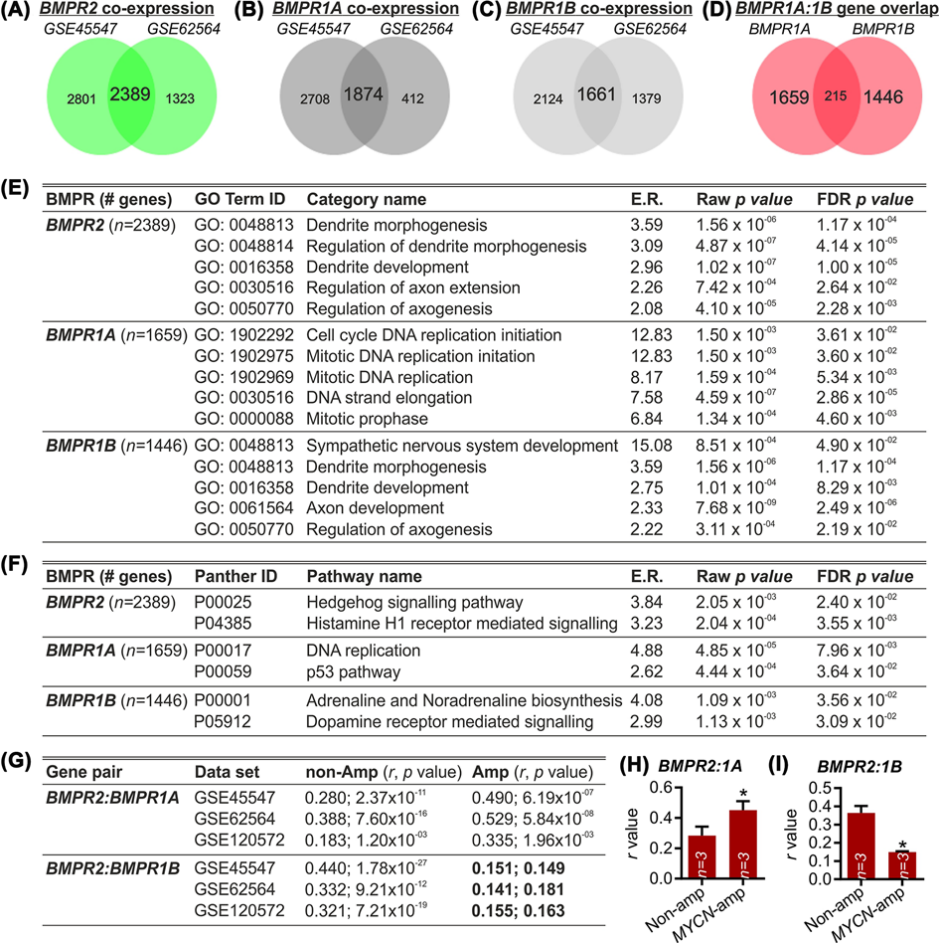
Specific BMPRs display distinct gene co-expression profiles enriched in genes associated with molecular pathways linked to NB proliferation or differentiation Venn diagrams generated using GeneVenn, showing the overlap in genes that display a Bonferroni-corrected significant positive co-expression pattern with (**A**) *BMPR2*, (**B**) *BMPR1A* or (**C**) *BMPR1B* in GSE45547 and GSE62564, as indicated. (**D**) Venn diagram of the overlap in *BMPR1A* and *BMPR1B* co-expressed genes, showing that the two receptors are associated with largely distinct groups of co-expressed genes. (**E**) Gene ontology (GO) and (**F**) Panther pathway enrichment analysis of the gene lists that were co-expressed with *BMPR2* (*n*=2389), *BMPR1A* (*n*=1659) and *BMPR1B* (*n*=1446) in both GSE45547 and GSE62564 data sets. (**G**) Table showing the correlation (*r*) and associated *P* values between *BMPR2* and *BMPR1A* or *BMPR1A* in non-*MYCN*-amplified (non-Amp) or in MYCN-amplified (Amp) NB cases in GSE45547, GSE62564 and GSE85047, as indicated. Graphs showing the mean ± SEM of the *r* value for (**H**) *BMPR2:BMPR1A* and (**I**) *BMPR2:BMPR1B* * *P*<0.05, vs. non-Amp; Student’s *t*-test; GO and Panther pathway analysis was carried out using tools available at www.geneontology.org. Gene correlation analysis was carried out using the R2 genomics analysis and visualization platform.

To investigate the signalling pathways that may underlie the distinct survival probabilities associated with expression of different BMPRs, we used gene co-expression analysis to identify all genes that displayed a positively correlated pattern of expression with distinct BMPRs in NB samples. The rationale for this approach is that correlated patterns of gene expression can reflect a functional association [35,36]. We identified from among all genes, those that had a significant positive correlation with the expression of *BMPR2* (*n* = 2389; Figure 3A), *BMPR1A* (*n* = 1874; Figure 3B) and *BMPR1B* (*n* = 1661; Figure 3C) in both data sets (GSE45547 and GSE62564)*.* We also compared the genes that were co-expressed with BMPR1A and with BMPR1B, and found that these two receptors were associated with largely distinct groups of genes (Figure 3D). We next performed a gene set enrichment analysis on these distinct gene lists for *BMPR2* (*n* = 2389), *BMPR1A* (*n* = 1659) and *BMPR1B* (*n* = 1446). For those genes that were co-expressed with *BMPR2* or *BMPR1B*, GO enrichment analysis revealed a statistically significant overrepresentation of genes within the multiple GO categories associated with neuritogenesis, including ‘dendrite development’ (GO:0016358) and ‘regulation of axogenesis’ (GO:0050770) (Figure 3E). These categories were not represented among BMPR1A co-expressed genes; in this case, GO enrichment analysis revealed a statistically significant overrepresentation of genes within the multiple GO categories associated with cell division and cell proliferation, including ‘Cell cycle DNA replication initiation’ (GO:1902292) (Figure 3E). Selected GO categories are shown in Figure 3E, with the full list available in Supplementary Tables S1–S3. We next sought to determine whether there was an overrepresentation in the list of BMPR co-expressed genes, of those belonging to specific Panther pathways. For *BMPR2* and *BMPR1B* co-expressed genes, there was a statistically significant overrepresentation of genes within Panther pathways associated with NB differentiation (Figure 3F). In contrast, in BMPR1A co-expressed genes there was a statistically significant overrepresentation of genes within Panther pathways linked to NB proliferation, such as ‘DNA replication’ and the ‘p53 pathway’ (Figure 3F). Selected GO categories are shown in Figure 3E, with the full list available in Supplementary Tables S4–S6. These data suggest that the reduction in *BMPR1B* and increase in *BMPR1A* in stage 4 NB, and in *MYCN*-amplified NB, may lead to a failure of differentiation and an increase in proliferation, respectively.

We next examined the coexpression patterns of *BMPR2* with *BMPR1A* and with *BMPR1B*, in stage 1 and stage 4 NB, and in non-*MYCN*-amplified or *MYCN*-amplified NB cases in the GSE45547 and GSE62564 data sets. To allow statistical analysis of any changes, we also added an additional data set, GSE120572 [32]. The rationale for doing this was that in a range of diseases, normal coexpression patterns tend to change, and broken or strengthened correlations can be used as an index of functional misregulation [37–39]. We found that the correlation between *BMPR2* and *BMPR1A* was strengthened in *MYCN*-amplified NB (Figure 3G,H). However, the correlation between *BMPR2* and *BMPR1B* was no longer significant in *MYCN*-amplified NB (Figure 3G) and was significantly reduced compared with non-*MYCN*-amplified NB (Figure 3I). Collectively, these data suggest a BMPR2:BMPR1B functional misregulation in *MYCN*-amplified NB, and that this may contribute to alterations in neuritogenesis that have been reported in NB cells [3].

### BMPR1B signalling promotes neurite growth in *MYCN*-amplified NB cells

**Figure 4:**
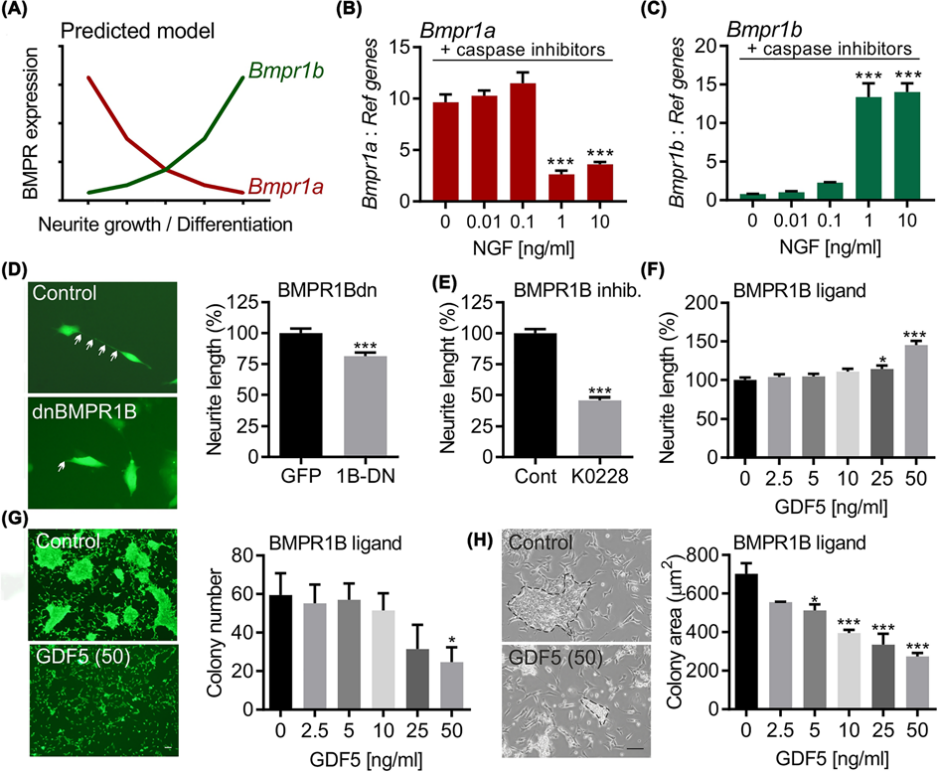
BMPR1B signalling promotes neurite growth in NB cells (**A**) Schema of a predicted model based on the NB data showing the predicted changes in type1 BMP receptor expression during sympathetic differentiation. Expression of transcripts for (**B**) *Bmpr1a* and (**C**) *Bmpr1b* in P1 mouse sympathetic neurons cultured with NGF at the indicated concentrations for 24 h to promote differentiation. Data are expressed relative to the levels of the geometric mean of three reference mRNAs: *Gapdh, Sdha* and *Hprt1*. Cultures were also treated with 25 μM of the pan-caspase inhibitor Boc-D-FMK. Data are mean  ±  SEM from  *n*=4 separate cultures. *** *P*<0.001 vs. control (0 ng/ml NGF); one-way ANOVA with post-hoc Dunnett’s test. Representative images and graph of neurite length of SK-N-BE(2) at 72 h post transfection with (**D**) a control plasmid (GFP) or a plasmid expressing dominant negative BMPR1B (dnBMPR1B), or (**E**) following treatment with 0.5 µM K02288, a BMPR1B inhibitor. (**F**) Graph of neurite length of SK-N-BE(2) at 72 h post treatment with GDF5 at the indicated concentrations. * *P*<0.05, *** *P*<0.001 vs. control (0 ng/ml GDF5); Student’s *t-*test for two groups or alternatively one-way ANOVA with post-hoc Dunnett’s test, as appropriate. Data are mean ± SEM of (**E**) 240 or (**F**) 180 individual cells per group from *n*=3 separate experiments. Representative images and graphs showing (**G**) the number of colonies and (**H**) colony area in SK-N-BE(2) cultures at 72 h post treatment GDF5 at the indicated concentrations. Data are mean ± SEM from *n*=3 separate experiments. * *P*<0.05, *** *P*<0.001 vs. control (0 ng/ml GDF5); one-way ANOVA with post-hoc Dunnett’s test.

*BMPR1A* co-expressed genes were found to be enriched in pathways linked to proliferation, and *BMPR1B* co-expressed genes to be enriched in those linked to neurite growth. This suggests a predicted model in which the expression of BMPR1A may be decreased, while the expression of BMPR1B may be increased during sympathetic neurite growth (Figure 4A). We tested this model by treating cultured P0 mouse SCG sympathetic neurons with NGF, a potent inducer of neurite growth in sympathetic neurons [40]. Cultures were also treated with 25 μM of pan-caspase inhibitor Boc**^_^**D**^_^**FMK, a well-established approach to prevent apoptosis at low concentrations of NGF [41,42]. Treatment with NGF at concentrations of 1 or 10 ng/ml resulted in a significant decrease in the expression of *Bmpr1a* (Figure 4B). In contrast, these concentrations of NGF resulted in significant increases in *Bmpr1b* expression (Figure 4C), while *Bmpr2* expression was unaffected (Supplementary Figure S1). This supports the hypothesis that distinct BMPRs are required for optimal neuritogenesis in NB cells. To test this directly, we use three complementary approaches. First, MYCN-amplified SK-N-BE(2) cells were transfected with a plasmid expressing a dominant negative (dn) form of BMPR1B generated by a single amino acid substitution (K to R) within the adenosine triphosphate binding site, which has been previously shown to reduce kinase activity dramatically [43,44] (Figure 4D). Second, these cells were treated with K02288, a BMPR and activin receptor inhibitor (Figure 4E), and neurite growth was measured at 72 h. Both dnBMPR1B (Figure 4E) and K02288 (Figure 4F) treatment led to significant reductions in neurite growth. Third, we treated SK-N-BE(2) cells with the BMP ligand GDF5, which has been shown to signal via BMPR1B but not BMPR1A [9,10]. We have previously shown that GDF5 increases pSmad1/5 signalling in SH-SY5Y cells [27] and in SCG neurons [20]. Here, we found that GDF5 treatment of SK-N-BE(2) cells increased pSmad1/5 levels (Supplementary Figure S2) and led to significant increases in neurite growth at 72 h (Figure 4F). We also found a similar result in non-MYCN amplified SH-SY5Y cells that were transfected with dnBMPR1B or treated with K02288 (Supplementary Figure S3A,B respectively). Moreover, the neurite growth-promoting effects of GDF5 were blocked by the K02288 inhibitor (Supplementary Figure S4). Moreover, GDF5 treatment for 72 h also reduced the number of colonies (Figure 4G) and the colony area (Figure 4H) in SK-N-BE(2) cultures. To confirm that both SK-N-BE(2) and SH-SY5Y cells expressed both BMPRs, we performed an analysis using open source transcriptome data (GSE: 90683R1) and found that both cell lines express both receptors but express BMPR1A at higher levels than BMPR1B (Supplementary Figure S5). Collectively, these data suggest that BMPR1B signalling is required to promote optimal neuritogenesis in *MYCN*-amplified NB cells.

## Discussion

Our analysis of the expression patterns of specific BMPRs in NB patient data sets showed strong correlations with disease stage and type. Specifically, the expression levels of both BMPR1B and BMPR2 were significantly lower at stage 4 NB, which is associated with a poor survival outcome, than at stage 1 NB, which has a better prognosis. The expression of BMPR1B and BMPR2 was also found to be lower in *MYCN*-amplified than in non-*MYCN*-amplified NB. *MYCN* amplification is associated with poor survival in NB [34], suggesting that reduced BMPR1B and BMPR2 expression may be a factor underlying poor survival outcomes for NB patients. In contrast, the expression of BMPR1A was significantly higher in stage 4 than in stage 1 NB, and was also higher in *MYCN*-amplified than non-*MYCN*-amplified NB, implying that this BMP receptor plays an opposing role to that of BMPR1B and BMPR2 on NB cell fate. In agreement, our analysis of associations between the expression of distinct BMPRs and survival probability in patient data sets showed that there are strong correlations between low BMPR2, low BMPR1B, high BMPR1A, as well as high MYCN, with poor survival probability in NB patients.

Reduced expression of BMPR1B has previously been found to be correlated with poor prognosis in breast cancer [45]. That study reported lower levels of BMPR1B expression in breast tumour samples compared with normal breast tissue samples, as well as reduced BMPR1B mRNA in samples taken from patients with predicted poor prognosis. Furthermore, down-regulation of BMPRIB resulted in increased proliferation of breast cancer cells *in vitro* [45]. Our finding that reduced BMPR2 expression was correlated with poor survival outcome is also consistent with a previous study, which reported a link between reduced BMPR2 expression and NB [21]. BMPR2 expression was significantly down-regulated in NB tissue samples, particularly in high-grade NB, and was inversely related to the expression of markers of NB differentiation [21]. Disruption of BMPR2 has also been found to promote metastasis of mammary tumours, suggesting that BMPR2 has tumour-suppressive function in mammary epithelial cells [46]. A study on mammary cell tumours in mice found that conditional knockout of the BMPR1A receptor delayed the onset of tumour formation, and extended cell survival [47]. That study also analysed human data sets and found a correlation between high BMPR1A gene expression and decreased survival, regardless of the molecular breast cancer subtype, and concluded that BMPR1A is a tumour promoter in human breast cancer [47]. Interestingly, a recent paper identified parallels between mammary cell tumorigenesis and neural crest lineage, by showing that SOX10 expression correlates with invasiveness and can elicit neural crest-like features in mammary tumours [48]. Along with our data, all of these findings suggest that reduced expression of BMPR1B and BMPR2, and/or increased expression of BMPR1A, may play a role in the determination of a proliferative cell fate, at least in some types of tumours.

Our analysis of the NB patient data sets showed that the expression of distinct BMPRs are associated with different survival outcomes in NB patients and suggest that the signalling pathways activated by these receptors may be involved in determining the fate of NB cells. We then proceeded to interrogate the signalling pathways that are associated with survival probability in these cells. Our gene co-expression analysis showed that high expression of BMPR1A was associated with proliferative cellular events, such as DNA replication and the p53 pathway. In contrast, high expression of BMPR1B and BMPR2 was associated with developmental cellular events, such as dendrite morphogenesis, axon development and sympathetic nervous system development. These data suggest that the reductions in BMPR1B and BMPR2, and the increase in BMPR1A, that are associated with both stage 4 NB and MYCN-amplified NB may combine to drive cells towards a fate involving failure of differentiation and promotion of proliferation. The balance between the expression profiles of BMPR1B and BMPR2 on one side, and BMPR1A and MYCN on the other, may be an important factor in deciding cell fate and state in NB.

Our analysis of the co-expression patterns of BMPR2 with BMPR1A and with BMPR1B, in three patient data sets, showed that in *MYCN*-amplified NB the correlation between BMPR2 and BMPR1A was strengthened, whereas the correlation between BMPR2 and BMPR1B expression was lost. This suggests a functional misregulation of BMPR2:BMPR1B binding in *MYCN*-amplified NB cells. It is possible that *MYCN* amplification may lead to defects in the formation of the BMPR2:BMPR1B receptor complex, which may in turn result in dysfunction of the downstream signalling pathways that are normally activated when BMP ligands bind to these receptors. It is known that there are defects in neuritogenesis in NB cells [3]. Since BMP signalling through the BMPRs plays a critical role in neuritogenesis in the developing nervous system (for review see [4]), it is possible that cell autonomous alterations in BMPR subtype expression within NB cells cause these cells to undergo oncogenic signalling responses to the BMP morphogens that would normally induce their neuronal differentiation.

To test the hypothesis that BMPR1B signalling promotes neurite growth in NB cells, we used two experimental approaches to block BMP–BMPR1B signalling, both of which resulted in significant reductions in neurite growth in SK-N-BE(2) and SH-SY5Y cells. In support of this, treatment of the MYCN-amplified SK-N-BE(2) NB cell line with the BMP family member GDF5, which signals through BMPR1B but not BMPR1a, promoted neurite growth. Thus, these data support the hypothesis that BMPR1B signalling is required to promote optimal neuritogenesis in NB cells.

In summary, the present study has shown that individual BMPRs have distinct expression patterns that are associated with different signalling pathways and survival outcomes in NB cells. This suggests that BMP ligands acting at distinct BMPRs play important roles in NB cell development and differentiation. In these cells, BMPR1A expression was associated with cell proliferation signalling pathways, whereas expression of BMPR1B and BMPR2 was associated with cellular differentiation and neurite growth. Furthermore, in NB patient data sets, poor survival outcomes were strongly correlated with reductions in BMPR1B and BMPR2 expression, and with high levels of BMPR1A expression. Finally, *MYCN*-amplification appears to be associated with a preferential BMPR2–BMPR1A coexpression pattern, which may result in a shift in intracellular signalling responses towards cell proliferation. By examining changes in signalling pathway components in different tumour cell types and stages, the oncogenic mechanisms underlying cancer progression can be elucidated.

## Supplementary Material

Supplementary Figures S1-S5

Supplementary Tables S1-S6

## Data Availability

The raw data supporting the conclusions of this manuscript will be made available by the authors, without undue reservation, to any qualified researcher.

## Abbreviations

BMP: bone morphogenetic protein
BMPR: bone morphogenetic protein receptor
NB: neuroblastoma
SCG: superior cervical ganglion
