# Supplementary material for: Association of distinct type 1 bone morphogenetic protein receptors with different molecular pathways and survival outcomes in neuroblastoma
Source: Neuronal Signal. 2020 Apr 23;4(1):NS20200006. doi: 10.1042/NS20200006 (PMC7366490; doi:10.1042/NS20200006)
Supplement: Supplementary Figures S1-S5 [file NS-2020-0006_supp.pdf]

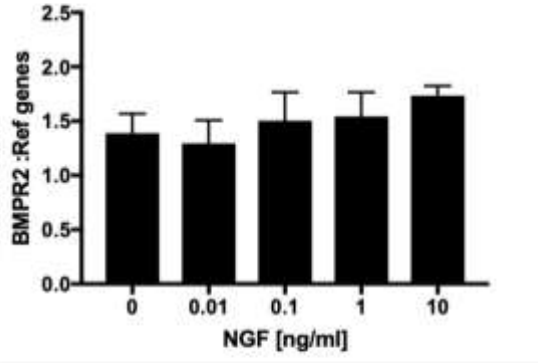

Supplementary Figure 1: Effect of increasing NGF concentrations on *Bmpr2* expression. Graph showing the expression of transcripts for *Bmpr2* in P1 mouse sympathetic neurons cultured with NGF, at the indicated concentrations, for 24 h. Data are expressed relative to the levels of the geometric mean ofexpression of transcripts for *Gapdh*, *Sdha* and *Hprt1*. Data are mean ± SEM from *n* = 4 separate cultures.

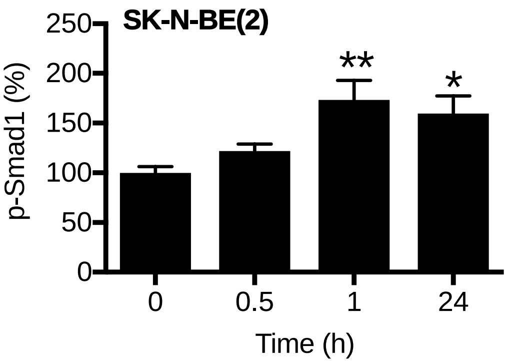

Supplementary Figure 2: Effect of GDF5 on pSmad1 in SK-N-BE(2) cells.
Graph showing the levels of pSmad1 as measured by ELISA in SK-N-BE(2) cells treated with 50ng/ml GDF5 for the indicated time points. Data are mean ± SEM from $n = 3$ separate cultures. *$p < 0.05$, ** $p < 0.01$ vs. control (0 h time point); one-way ANOVA with *post-hoc* Dunnett's test.

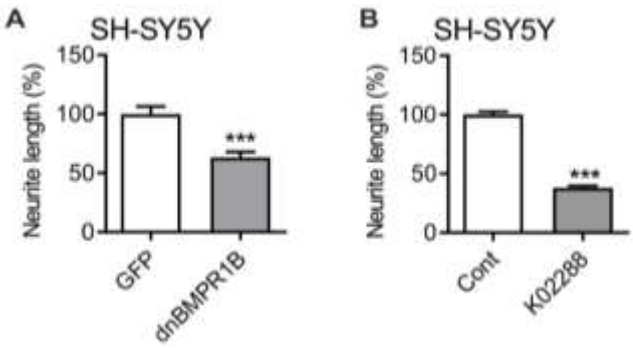

Supplementary Figure 3: Inhibition of BMPR1B signalling leads to a reduction in neurite growth in SH-SY5Y cells.

(A, B) Graphs of neurite length of SH-SY5Y cells at 72 h post transfection with (A) a control plasmid (GFP) or a plasmid expressing dominant negative BMPR1B (dnBMPR1B), or (B) following treatment with 0.5 µM K02288, a BMPR1B inhibitor. (*** $p < 0.001$ vs. control; Student's *t*-test).

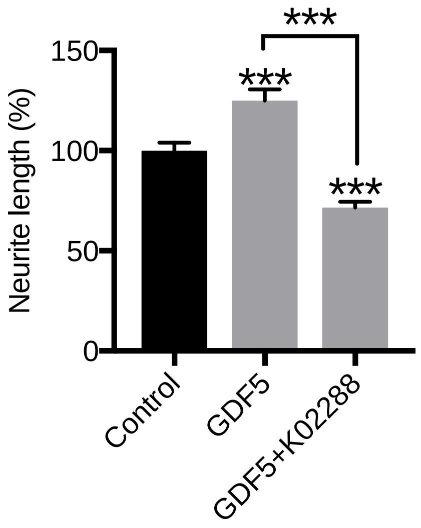

Supplementary Figure 4: Effect of K02288 on GDF5-promoted neurite growth.
Graphs of neurite length of SH-SY5Y cells at 72 h post treatment with GDF5 with or without 0.5 µM K0228, a BMPR1B inhibitor. (*** p < 0.001 vs. control; One-way ANOVA, *post-hoc*Tukeys test).

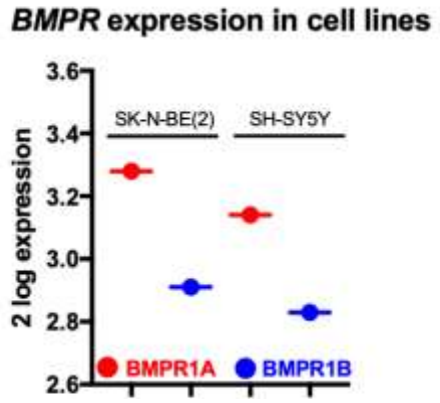

Supplementary Figure 5:Expression of transcripts for *BMPR1A* and *BMPR1B* in SK-N-BE(2) and SH-SY5Y cells.

Graph showing log 2 expression values of *BMPR1A* and *BMPR1B* in SK-N-BE(2) and SH-SY5Y cells using open source transcriptome data (GSE: 90683R1) and analysed in the R2 genomics analysis and visualisation platform.
